# The Mediating Effect of Physical Fitness and Dietary Intake on the Relationship of Physical Activity with Body Composition in High School Students

**DOI:** 10.3390/ijerph19127301

**Published:** 2022-06-14

**Authors:** André Bento, Luis Carrasco, Armando Raimundo

**Affiliations:** 1 Comprehensive Health Research Centre (CHRC), Department of Sport and Health, School of Health and Human Development, University of Évora, 7004-516 Évora, Portugal; ammr@uevora.pt; 2 BIOFANEX Research Group, Department of Physical Education and Sport, University of Seville, 41004 Seville, Spain; lcarrasco@us.es

**Keywords:** exercise, health, fitness, youth

## Abstract

We aimed to investigate the relationship between physical activity (PA) and health-related physical fitness (PF) in adolescents and analyze if the associations of PA with body composition (BC) in adolescents are mediated by physical fitness or energy intake (EI). The participants were 236 adolescents (140 girls 16.1 ± 0.92 years). Cardiorespiratory fitness (CRF) was assessed using the Yo-YoITL1, and the push-up test was used to evaluate strength. BCs were measured on an electrical weight scale. Triaxial accelerometers were used to determine PA levels and moderate-to-vigorous PA (MVPA) levels. EI was estimated with a validated questionnaire. Mediation effects were estimated using bootstrapped 95% confidence intervals and were deemed significant if zero was not included in the intervals. The mediation analysis revealed an indirect effect of MVPA only through PF on BC, specifically through CRF on body fat (B = −0.0146, 95% BootCI (−0.0219; −0.0076)) and on lean body mass (B = 0.0096, 95% BootCI (0.0049; 0.0152)), as well as through upper body strength on body fat (B = −0.012, 95% BootCI (−0.0171; −0.0072)) and on lean body mass (B = 0.0059, 95% BootCI (0.003; 0.0095)). These results suggest that PA of at least a moderate intensity is relevant to BC and health-related PF in adolescents, regardless of the EI.

## 1. Introduction

Low cardiorespiratory fitness (CRF), measured by maximal oxygen consumption (VO_2_max), is a powerful predictor of all-cause mortality and morbidity in young people [1]. Despite the numerous benefits of regular physical activity (PA), Western children and adolescents spend too much time in sedentary behaviors, which is worsening every decade [2,3,4,5]. Moreover, adolescents tend to become more inactive as their age increases [6]. Short leisure time, reduced access to facilities, as well as low motivation to engage in physical activities, are frequently reported barriers to poor adherence to exercise programs [7,8,9]. In any case, higher amounts of sedentary behavior are associated with increased adiposity, as well as poorer cardiometabolic health and fitness in children and adolescents [10].

In modern society, with the economic development and changes in dietary structure, high-calorie foods are more prevalent among adolescents, leading to a significant increase in the obesity rate of the youngest [11] and to the promotion of a positive energy balance. Because of its direct relationship to the long-term gain or loss of adipose tissue and changes in metabolic pathways, the difference between energy intake and expenditure has become of great interest since it was recognized that overweightness and obesity are major risk factors for many other health conditions [12]. While objective metrics for assessing energy expenditure or PA exist [13,14], there are none for assessing energy intake. Measures of energy intake, although not being useful for assessing energy balance, as well as PA continue to play other important roles in epidemiologic studies and in monitoring population trends. Diet combined with PA leads to higher weight loss and is more effective at keeping lean body mass, resulting in a more desirable influence on overall body composition than diet alone [15].

Energy intake patterns and PA behaviors are influenced by a complex interaction of these factors. The focus of PA research has expanded to address the potential negative effects of sedentary behavior on energy balance [12]. Higher levels of PA have been demonstrated to reduce the weight gain associated with increased energy density, and it appears that at low levels of PA, an effective appetite suppression to maintain energy balance is compromised [16]. Environmental factors should be the primary objectives of epidemic intervention efforts, as they are modifiable, although genetic factors may play a role in affecting individuals’ susceptibility to developing obesity [12].

The World Health Organization (WHO) stated that this population should achieve at least an average of 60 min per day of moderate-to-vigorous PA (MVPA) and must limit sedentary time, and, as good practice, recommend that doing some physical activity is better than doing none [10]. Every move counts, says WHO. Notwithstanding, the limited number of people willing to engage in MVPA and the high attrition of those who participate [17], the evidence shows a high effectiveness of MVPA to reduce mortality, even considering a long lifespan [18]. In modern society, it is unlikely that individuals will ever return to the high average PA levels of the past, and time-efficient interventions have a prominent role. Intervention and implementation research is needed to learn about social and physiological changes, in particular among the youngest in school-based settings, therefore allowing us to identify possible indicators to guide national strategies in promoting healthy lifestyles in young people.

According to Bond et al. [19], the time spent in high-intensity activities is the most important factor in promoting vascular health and autonomic cardiac modulation. There is a lack of studies evidencing the association between PA and physical fitness development in children and adolescents [20]. Additionally, there is an important lack of data on diet, PA, and adiposity in most parts of the world [12]. To the authors’ knowledge, no previous study has examined the mediating role of physical fitness or energy intake in PA and body composition relationships in Physical Education classes (PEC). Based on the need and importance to increase the knowledge about the relationship between PA and health-related body composition, we aimed to analyze the level of PA, energy intake, and fitness in a sample of the Portuguese adolescent population. As a result, the primary goal of this research was to examine the indirect (physical fitness or energy intake-mediated) effects of PA on adolescents from the 10th to 12th grades on body composition (body fat and lean body mass). More precisely, investigating as to how these variables are associated allowed us to identify possible indicators to guide national strategies in promoting healthy lifestyles in young people. Identifying the pathways or systems that mediate the effects of PA on physical fitness is essential for the development of successful PEC interventions. 

## 2. Materials and Methods

### 2.1. Study Design

Data were retrieved from the baseline assessment of a randomized controlled trial investigating the effects of High-Intensity Interval Training (HIIT) in High School PEC. The Ethics Committee of the University of Évora (doc. 19017) approved this study, which was registered on ClinicalTrials.gov (ID: NCT04022642). This trial was conducted in accordance with the Declaration of Helsinki on Human Research.

### 2.2. Participants

Two public schools in the Portuguese city of Beja were invited to participate. Before participating, the school administrator and parents signed a consent form. The researchers met with the school principal after receiving an invitation and provided information on the whole project. After accepting to participate, 236 adolescents from the 10th to 12th grades (96 boys and 140 girls, mean age 16.1 years, SD = 0.92) and their parents were informed of a full description of the scientific background, objectives, and safety. Students were ineligible if they did not provide parental consent to participate, had physical limitations, or revealed intellectual disabilities.

### 2.3. Measurements

The Principal Investigator assessed PA and physical fitness (CRF, upper-body strength, and body composition) at the schools participating in the study. When possible, participants’ body composition and body mass assessments were performed in the presence of a same-sex research team. Before the evaluation, the Principal Investigator gave a brief verbal description and an explanation of each fitness test.

#### 2.3.1. Physical Activity

Triaxial accelerometers were used to measure PA levels and sedentary time for seven days, as recommended in a recent systematic review [14]. The ActiGraph wGT3X-BT accelerometer, which was connected to an elasticized belt and positioned on the right side of the participant’s hip, was to be worn on their waist for 24 h per day (and only removed during shower time or swimming activities). For valid records, at least three weekdays and one weekend day were required to be reported. PA was calculated (i.e., mean minutes per day) using existing thresholds for categorizing physical activity into sedentary behavior, as well as light, moderate, and vigorous intensity.

#### 2.3.2. Physical Fitness

The Yo-Yo Intermittent Endurance Test level one was used to test CRF. This test has previously been demonstrated to be valid and accurate for assessing aerobic fitness and intermittent high-intensity endurance in children aged 9 to 16 [21]. It also consists of incremental shuttle running starting from the speed of 8 km·h^−1^ until exhaustion. The maximum running speed is 14.5 km·h^−1^. Each shuttle run consists of 2 × 20 m interspersed by 10 s of active recovery (slow jog or walk) for a short 2.5 m shuttle. There are multiple shuttle runs within each speed stage. A pre-recorded audio track dictates the running speed. By the time each audio is played, participants must have crossed the 20 m line. The test is over if the participant cannot maintain the appropriate speed for the second time during the shuttle running bout. Using the following equation, the total number of laps were utilized to estimate maximal aerobic capacity (VO_2_max): IRT1 distance (m) × 0.0084 + 36.4 (mL kg^−1^·min^−1^) [22]. During testing, telemetric heart rate (HR) was used to monitor HR. The maximum HR was considered to be represented by the peak HR observed during the test [23]. FITNESSGRAM thresholds were used to identify “low fitness” levels [24].

Upper body strength was assessed using the push-up test (FITescola^®^; [25]). The test started with the participant’s hands and feet touching the floor, and their body in a plank position, with their feet apart and their hands positioned below the shoulder line. With the body straight (without arching up or slumping down) and only the hands and toes touching the floor the participants should lower the body until forming a 90° angle between the arm and the forearm and then return to the starting position. This action was repeated with a previously defined cadence of 20 push-ups per minute. Cooper Institute thresholds were used to identify “low fitness” levels [26].

#### 2.3.3. Body Composition

Participants’ body composition and body mass were measured to the nearest 0.1 kg in light sportswear on a bioelectrical impedance scale (Tanita MC −780, Tokyo, Japan), and height was measured to the nearest 1 mm using a portable stadiometer (Seca 213 Portable Height Measuring Rod Stadiometer). Body composition measurements (body fat, lean body mass and basal metabolic rate) were performed through a bioelectrical body impedance analysis (BIA). Female adolescents did not have a menstrual period during examinations, and measurements were taken without metal items (earrings, belts, coins). To ensure the proper hydration for BIA testing, participants were asked to follow the following pre-test conditions: there should be no vigorous exercise or caffeine or alcohol consumption within 12 h of the test [27]. Body fat (BF) percentage thresholds were identified in relation to metabolic syndrome status [28]. Both weight and height were measured twice to reduce the risk of measurement error. BMIs were calculated using the standard formula (weight (kg)/height (m^2^)). Age-specific and sex-specific BMI z-scores were calculated and used to classify participants into weight categories [29].

#### 2.3.4. Dietary Registration

Daily dietary intake was estimated with a validated self-reported food frequency questionnaire [30], which lists food types and meals that are typical of a Portuguese diet. A semiquantitative food frequency questionnaire from the previous month was used to assess daily dietary intake, which included 82 food or beverage categories and a frequency section with nine possible responses ranging from never to six or more times per day.

### 2.4. Statistical Analysis

Descriptive statistics were used to characterize the subjects and exercise test results. All variables were assessed for normality using the Kolmogorov–Smirnov test. A bivariate analysis was performed, using the parametric Pearson correlation coefficient or the nonparametric Spearman correlation coefficient (r_s_), in order to indicate the strength of the association between variables. Interpretation of correlation coefficients was as follows: r ≤ 0.49 weak relationship; 0.50 ≤ r ≤ 0.74 moderate relationship; r ≥ 0.75 strong relationship [31]. To examine whether physical fitness or energy intake mediated the relationship of MVPA with body composition, MVPA was used as a predictor in distinct models for each outcome, and physical fitness or energy intake were used as a mediator.

**Figure 1:**
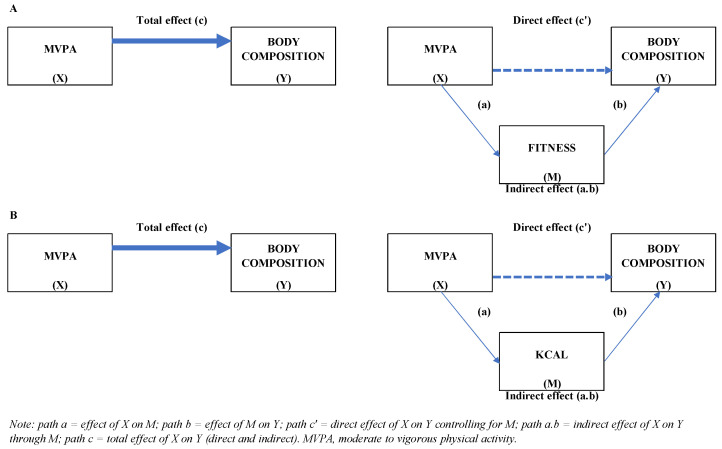
Conceptual models of mediation analysis: indirect effect through fitness (**A**) or kcal (**B**).

Figure 1 illustrate the overall mediation models used in the analysis, a nonparametric sampling procedure, bootstrapping, has been advocated to obtain percentile-based confidence limits [32,33,34]. This procedure provides the total and specific indirect effects (through the proposed mediator: fitness or energy intake) of the predictor (MVPA) on the outcomes (body composition). SPSS macro developed by Preacher and Hayes [33] (PROCESS version 4.0) was used to test mediation. The indirect impact was calculated using 10,000 bootstrap samples for the bootstrap confidence intervals (CI), corrected for bias. An indirect effect is considered to be statistically significant if the CI established (95% CI, 95% BootCIs) does not include 0. If the CI contains the value 0, the null hypothesis demonstrates that the indirect effect equals 0, i.e., there is no association between the variables considered. Finally, unstandardized (B) and completely standardized effects (β) were used to describe the indirect effects. All *p*-values were two-tailed, and values below 0.05 were considered to indicate statistical significance. All statistical analyses were performed with the Statistical Package for the Social Sciences v.24 (SPSS Inc., Chicago, IL, USA).

## 3. Results

Data for 236 students (59.3% female) aged 16.1 ± 0.92 years that presented valid accelerometry data were available for analysis (Table 1). Overall, the bivariate analyses demonstrate consistent small to medium significant relationships between the variables. Regarding body composition, despite 82% of the students presenting with a normal weight, 33% are at high risk according to their body fat. Data from physical fitness assessment show that most students were at a low CRF level (71%), and only 23% showed a low muscular fitness level. Triaxial accelerometers data revealed that participants spent an average of 842.4 min per day on sedentary behaviors, 139.6 min per day on light PA, 23.0 min per day on moderate PA, and 13.6 min per day on vigorous PA. Boys had more time on vigorous PA than girls (*p* < 0.001). Only 8% of the students performed the recommended minutes of MVPA (at least 60 min per day).

Regarding body composition and physical fitness, the associations among the study variables showed less correlations for PA below MVPA. Regarding the risk thresholds presented in Table 1, bivariate correlation results showed, for body fat, a negative correlation with energy intake (ρ = −0.146, *p* < 0.05), CRF (ρ = −0.790, *p* < 0.01), upper body muscle force (ρ = −0.687, *p* < 0.01), vigorous PA (ρ = −0.400, *p* < 0.01), and MVPA (ρ = −0.341, *p* < 0.01). Thus, regarding physical fitness, CRF and strength, respectively, they showed positive correlations with energy intake (ρ = 0.269, *p* < 0.01; ρ = 0.256, *p* < 0.01), basal metabolic rate (ρ = 0.383, *p* < 0.01; ρ = 0.314, *p* < 0.01), vigorous PA (ρ = 0.482, *p* < 0.01; ρ = 0.400, *p* < 0.01), and MVPA (ρ = 0.347, *p* < 0.01; ρ = 0.310, *p* < 0.01), and a negative correlation between CRF and sedentary behavior (ρ = −0.139, *p* < 0.05). Light or moderate PA did not reveal any correlation with energy intake, physical fitness, or body composition. Sedentary behavior showed a negative correlation with energy intake (ρ = −0.136, *p* < 0.05), lean body mass (ρ = −0.177, *p* < 0.01), and basal metabolic rate (ρ = −0.144, *p* < 0.05). Correlations among the study variables are presented in Table 2.

**Figure 2:**
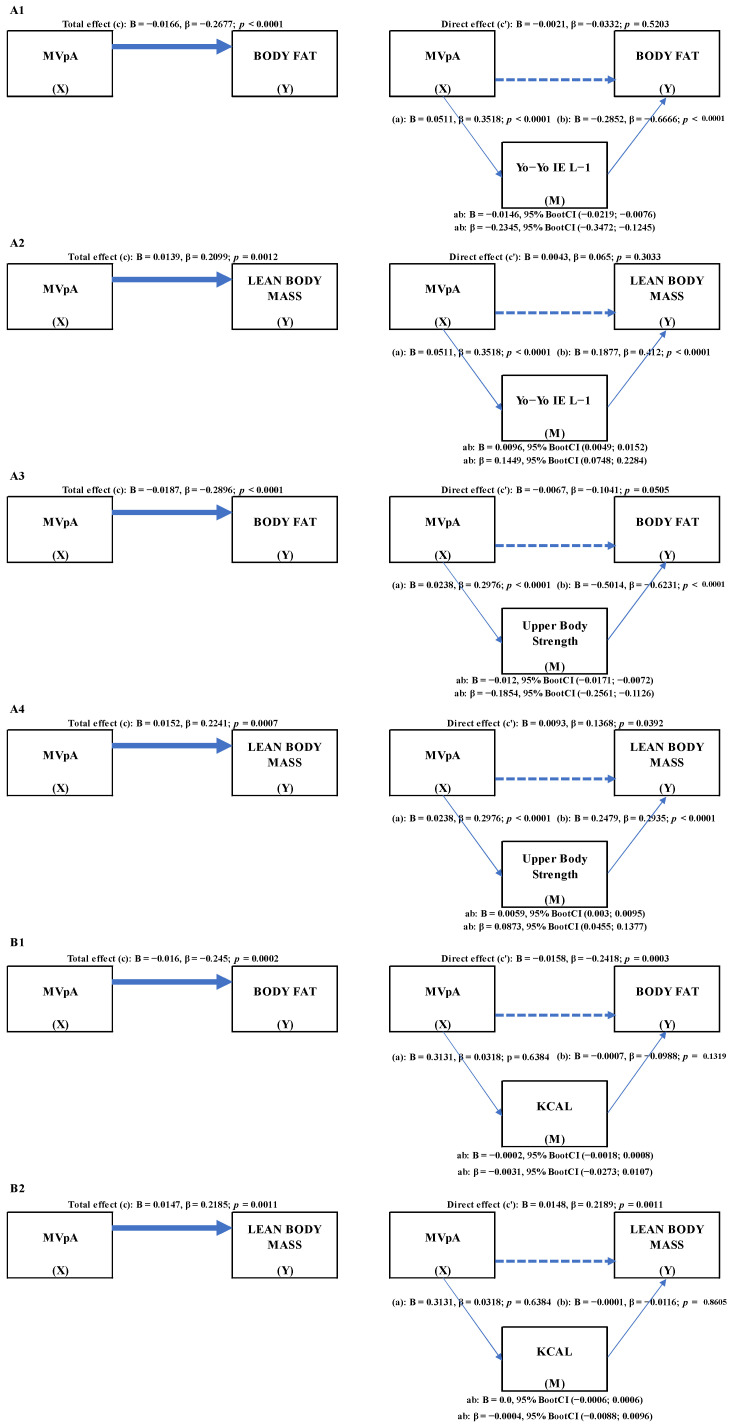
Mediation model A showing the effects (total, direct, and indirect) of MVPA on body composition variables. Mediation model B showing the effects (total, direct, and indirect) of MVPA on body composition variables. Abbreviations: MVPA, moderate-to-vigorous physical activity; IE L−1, Intermittent Endurance Test level one.

Figure 2A1–A4 show the results of the mediation analysis for each outcome according to Conceptual Model A (Figure 1). The total effect (*path c*) was significant on all models mediated through physical fitness or energy intake. Mediation through physical fitness does not include zero for any outcomes in the 95% BootCIs, revealing a significant indirect effect of MVPA, through physical fitness, on both the components of body composition, specifically for CRF on body fat (B = −0.0146, 95% BootCI (−0.0219; −0.0076)) and on lean body mass (B = 0.0096, 95% BootCI (0.0049; 0.0152)). This was also seen specifically for upper body strength on body fat (B = −0.012, 95% BootCI (−0.0171; −0.0072)) and on lean body mass (B = 0.0059, 95% BootCI (0.003; 0.0095)). Models A2 and A4 had a positive indirect effect since both *path a* and *path b* were positive, implying that high MVPA values improve physical fitness, which improves lean body mass. Because *path a* was positive and *path b* was negative in models A1 and A3, the indirect effect was negative. As a result, the indirect impact revealed that high MVPA levels promote physical fitness, which leads to a reduction in body fat. The completely standardized indirect effect indicated the largest indirect effect of MVPA through CRF on body fat (β = −0.2345, 95% BootCI (−0.3472; −0.1245)), followed by the indirect effect of upper body strength (β = −0.1854, 95% BootCI (−0.2561; −0.1126)), the indirect effect of CRF on lean body mass (β = 0.1449, 95% BootCI (0.0748; 0.2284)), and the indirect effect of upper body strength (β = 0.0873, 95% BootCI (0.0455; 0.1377)). Except for the influence of upper body strength on lean body mass, MVPA had no direct effects (*path c′*) on any outcomes.

According to conceptual model B (Figure 1), Figure 2B1,B2 show the findings of the mediation analysis for each outcome. The 95% BootCIs for any outcomes mediated through energy intake contained zero, indicating that MVPA has a non-significant indirect effect on both components of body composition, specifically body fat (B = −0.0002, 95% BootCI (−0.0018; 0.0008)) and lean body mass (B = 0, 95% BootCI (−0.0006; 0.0006)).

## 4. Discussion

These results provide support that youth and adolescents should do regular vigorous-intensity activity to improve CRF and muscular fitness. Despite high-calorie foods promoting a positive energy balance and leading to a significant decline in the body composition of the youngest [11], we found a non-significant indirect effect of MVPA for energy intake on both components of body composition. Moreover, we found a negative association between adiposity and energy intake and a moderate positive correlation between lean body mass and energy intake (ρ = −0.146, *p* < 0.05; ρ = 0.642, *p* < 0.01, respectively). There is an important lack of data on diet, PA, and adiposity in most parts of the world; for these reasons, there is no systematic information on the determinants of the divergent trends in body composition in children and adolescents, be it on food environments and behaviors or on policies that affect them [35]. Additionally, we found a negative association between sedentary behavior and energy intake and a moderate positive correlation between MVPA and energy intake, respectively (ρ = −0.136, *p* < 0.05; ρ = 0.739, *p* < 0.01).

The major findings of our study imply that MVPA has a positive indirect effect on body composition through physical fitness. These findings highlight the need of regular MVPA for maintaining or improving physical fitness, which has a positive impact on body composition. In children and adolescents, higher rates of sedentary behavior are associated with increased adiposity, as well as poorer cardiometabolic health and fitness [10]. In our study, the time spent doing light or moderate PA did not reveal any correlation with body composition. Even when the adolescents reported a higher time spent in sedentary behavior, their body fat did not show a positive correlation. PA is reported to be associated with adiposity, and higher levels of activity may be associated with healthy weight status; moreover, recent evidence reaffirms that increased PA improves CRF and muscular fitness [20]. In our study, we only found a positive association between adiposity and PA when adolescents reported a higher time spent in vigorous PA or combined vigorous plus moderate intensity, and in both dimensions of physical fitness: CRF and muscular fitness. 

Low CRF is a powerful predictor of all-cause mortality and morbidity in young people [1]. A recent meta-analysis reported that school-based PA programs were associated with improvements in diastolic blood pressure and fasting insulin when compared with non-PA interventions [36]. We found a strong negative correlation between CRF and body fat. Also, a positive association between CRF and PA was observed when adolescents reported a higher time spent in vigorous PA or combined vigorous plus moderate intensity. However, the time spent in light or moderate PA was not correlated with CRF. According to Bond et al. [19], the time spent in high-intensity activities is the most important factor in promoting vascular health and autonomic cardiac modulation. There is a lack of studies evidencing the association between PA and physical fitness development in children and adolescents [20]. We found a strong negative correlation between muscular fitness and body fat. Additionally, a positive association between muscular fitness and PA was observed when adolescents reported a higher time spent in vigorous PA or combined vigorous plus moderate intensity PA. Children and adolescents have expressed a clear preference for time efficiency and pleasure of MVPA, and its nature seems to reflect the activities traditionally observed in childhood [2,3,37]. Furthermore, adolescents seem to be more enthusiastic about resistance training, whereas aerobic training is found to be boring [38]. The lack of negative associations found in recent studies applying intense interventions are encouraging [39,40]; other studies, aside from aerobic and resistance training groups, included a variety of activities to enhance the motivation and appeal of exercise to the interest of older adolescents, thereby improving aspects of adolescents’ cardiometabolic health [41], fitness, and body composition, despite using the lowest dose of exercise among the groups [4].

Notwithstanding the impact that PA has on physical fitness and that both variables have on body composition, these variables (i.e., PA and physical fitness) were examined separately. Several studies have shown that physically active adolescents have a better body composition when compared to inactive adolescents [10,20]; the mechanisms by which PA may cause improvements in body composition are still unknown. According to our findings, the favorable effect of PA on body composition could be mediated by improvements in physical fitness, according to our findings. In line with our hypothesis, we observed a significant and positive indirect influence of MVPA on body composition through physical fitness. The largest indirect effect of MVPA through physical fitness was observed through CRF on body fat, followed by an indirect effect through upper body strength, an indirect effect through CRF on lean body mass, and an indirect effect through upper body strength. Overall, these findings suggest that accumulating MVPA on a daily basis promotes physical fitness, which improves body composition in adolescents.

The mediation mechanism assumes that the independent variable influences the mediator, and the mediator affects the dependent variable. So, the independent variable’s total effect is divided into indirect effects through a mediator. In our work, we used this statistical method to test if physical fitness and energy intake were mediators of the relationship of MVPA with body composition. In this study, our mediation analyses indicated that adolescents with a higher daily accumulation MVPA showed better physical fitness and therefore an improved body composition. This research adds to the existing literature by examining the indirect effect of PA on body composition in adolescents via physical fitness. It is critical to define and measure the mechanisms through which PA may be linked to body composition in order to improve recommendations and interventions. This is because Investigating mediators can aid in identifying critical aspects that require more attention in order to enhance outcomes.

Despite the novelty and interest of our findings, some limitations must be addressed. First, the use of a cross-sectional design is limiting, which prevents the determination of the temporality of the effect of MVPA, physical fitness, and energy intake on body composition; additionally, the inference of causality from our hypothesized path models is limited by the use of cross-sectional data. Second, our sample was limited to adolescents from the 10th to 12th grades from a public school in the city of Beja (Portugal), concluding that our findings cannot be applied to other populations. However, our selected bootstrapping method has strong statistical power and is considered a useful tool for avoiding Type I errors [32]. Third, using accelerometry to assess PA does not provide inferences about the form of physical activity undertaken. Furthermore, accelerometers do not detect all activities that may benefit physical fitness and body composition. The higher the intensity of PA, however, the more likely fitness and body composition adjustments are. To discover the specificities of PA required for the maintenance or improvement of physical fitness and body composition in adolescents, more intervention studies employing real-life scenarios are needed. Finally, other mediator variables may contribute to the links between PA and body composition. Measures of energy intake and expenditure are not precise enough, and the quality of the diet may exert its effect on energy balance through complex hormonal and neurological pathways that influence satiety, and, possibly, through other mechanisms [12].

## 5. Conclusions

The present study examined the relationship between PA, physical fitness, energy intake, and health-related body composition in adolescents. The aim of this study was to assess if MVPA had an indirect effect on body composition through physical fitness or calorie intake. Total sedentary time was not associated with health outcomes (physical fitness and body composition); nonetheless, the time in MVPA has been positively associated with fitness and body composition. Regular MVPA is indirectly associated with improvements in body composition in adolescents when using physical fitness as a mediator, but not energy intake. To our knowledge, this is the first study to use a mediation model to investigate the indirect influence of MVPA on body composition in older adolescents (through physical fitness or energy intake). Our findings suggest that MVPA is beneficial to adolescents’ health by promoting physical fitness maintenance or improvement; however, more research is needed to corroborate these findings. Moreover, exercise protocols that result in short-term physiological health improvements are of interest to physical education teachers, as well as to rehabilitation, health, and exercise professionals. The results of this study emphasize the importance of new strategies in PEC with acute vigorous-intensity activities, as well as those that strengthen muscle, which retain their health-enhancing effects and satisfy the adolescents’ desire for enjoyment and variety. The idea that public health gains will be greater if we help the least active become more active is being challenged. In a modern society, it is unlikely that individuals will ever return to the high average PA levels of the past. This study highlights that time-efficient interventions have a preeminent role.

## Figures and Tables

**Table 1:** Descriptive statistics of study variables (mean ± SD).

Title 1	Title 2
Anthropometric (n)	236 (f = 140)
Body Fat (%)	25.0 (7.8)
High metabolic syndrome risk	33%
Lean Body Mass (kg)	45.3 (8.7)
BMI (kg m^−2^)	22.0 (3.7)
Thinness (<−2 SD)	2%
Normal-weight	82%
Overweight (>+1 SD)	10%
Obesity (>+2 SD)	6%
Basal Metabolic Rate (Kcal)	1531.6 (249.3)
Dietary Registration (n)	236 (f = 140)
Daily Kcal	2129.3 (1170.1)
Cardiorespiratory Fitness (n)	235 (f = 139)
Yo-Yo IE L-1 (laps)	23.7 (18.2)
“low fitness” level	71%
Muscular Fitness (n)	223 (f = 134)
Push-ups (reps)	15.3 (9.7)
“low fitness” level	23%
Physical Activity (n)	236 (f = 140)
Sedentary (min day^−1^)	842.4 (161.5)
Light (min day^−1^)	139.6 (44.0)
Moderate (min day^−1^)	23.0 (9.5)
Vigorous (min day^−1^)	13.6 (11.1)
Recommended MVPA	8%

BMI: Body mass index; MVPA: moderate to vigorous physical activity.

**Table 2:** Correlations among study variables.

		1	2	3	4	5	6	7	8	9	10	11
1.	Body Fat	x										
2.	Lean Body Mass	−0.273 ^†^	x									
3.	BMI	0.556 ^†^	0.548 ^†^	x								
4.	Basal metabolic rate	−0.258 ^†^	0.977 ^†^	0.548 ^†^	x							
5.	Daily Kcal	−0.146 *	0.642 ^†^	0.445 ^†^	0.615 ^†^	x						
6.	Yo-Yo IE L−1	−0.790 ^†^	0.381 ^†^	−0.274 ^†^	0.383 ^†^	0.269 ^†^	x					
7.	Upper body strength	−0.687 ^†^	0.317 ^†^	−0.238 ^†^	0.314 ^†^	0.256 ^†^	0.727 ^†^	x				
8.	Sedentary	0.092	−0.177 ^†^	−0.044	−0.144 *	−0.136 *	−0.139 *	−0.114	x			
9.	Light	−0.020	0.039	0.062	0.060	0.433 ^†^	−0.029	0.003	0.272^†^	x		
10.	Moderate	−0.101	0.073	0.046	0.059	0.521 ^†^	0.072	0.078	0.154 *	0.567 ^†^	x	
11.	Vigorous	−0.400 ^†^	0.352 ^†^	0.009	0.339 ^†^	0.670 ^†^	0.482 ^†^	0.400 ^†^	0.012	0.290 ^†^	0.428 ^†^	x
12.	Daily MVPA	−0.287 ^†^	0.267 ^†^	0.046	0.242 ^†^	0.739 ^†^	0.347 ^†^	0.310 ^†^	−0.113	0.380 ^†^	0.740 ^†^	0.833 ^†^

BMI: Body mass index; MVPA: moderate to vigorous physical activity; * Correlation is significant at the 0.05 level (2-tailed); ^†^ Correlation is significant at the 0.01 level (2-tailed).

## Data Availability

The datasets generated during and/or analysed during the current study are not publicly available due to participants privacy protection but are available from the corresponding author on reasonable request.
